# Successful large-scale production of fruiting bodies of *Laetiporus sulphureus* (Bull.: Fr.) Murrill on an artificial substrate

**DOI:** 10.1007/s11274-012-1230-z

**Published:** 2012-12-11

**Authors:** Małgorzata Pleszczyńska, Adrian Wiater, Marek Siwulski, Janusz Szczodrak

**Affiliations:** 1 Department of Industrial Microbiology, Maria Curie-Skłodowska University, Akademicka 19, 20-033 Lublin, Poland; 2 Department of Vegetable Crops, Poznań University of Life Sciences, Dąbrowskiego 159, 60-594 Poznan, Poland

**Keywords:** *Laetiporus sulphureus*, Fruiting body, Intensive cultivation

## Abstract

*Laetiporus sulphureus* is an edible wood-rotting basidiomycete fungus whose fruiting bodies contain substances with verified therapeutic evidences and large amounts of α-(1 → 3)-glucan which is used as an effective inducer of microbial α-(1 → 3)-glucanases. However, production of mature fruiting bodies of this species under artificially controlled conditions has not been reported until now. Here, we provide the first report of successful initiation and development of *L. sulphureus* fruiting bodies in large-scale experiments. Twelve *Laetiporus* strains were isolated from a natural habitat. A synthetic log production system with a substrate composed of a mixture of sawdust enriched with organic and inorganic additives was developed. It was found that shocking the fungus mycelium with cold water or low temperature was the only suitable method for forced fruiting of *L. sulphureus* strains. Primordia of two strains were initiated already after 5–6 days from induction, and after another 2 days, they began to develop into fruiting bodies. Carpophores appeared fastest on substrates with high organic supplementation (40–45 %) and a low moisture content (40 %). The resulting mature fruiting bodies reached a weight of 200–300 g. The method of cultivation presented in this paper opens the way to commercial production of this valuable basidiomycete.

## Introduction

*Laetiporus sulphureus* (Bull.: Fr.) Murrill belonging to *Basidiomycotina*, *Aphyllophorales, Polyporaceae* is a parasitic and saprobic fungus growing alone or more typically in large clusters on decaying logs, stumps and trunks of many deciduous and coniferous tree species. It is widely distributed in Europe, Asia, and North America. Its striking yellowish or orange-colored shelf-like fruiting bodies appear usually in summer and fall, but very often are not formed until years after the fungus is well established, so when present, they indicate significant internal defect. *L. sulphureus* causes a reddish brown cubical heart rot, with thin areas of white mycelium visible in the cracks of wood (Gilbertson and Ryvarden 1986).

Due to its special fragrance and texture, *L. sulphureus* has been used for many years in oriental cultures as nutritional food. Besides food, fruiting bodies of *L. sulphureus* are a source of bioactive compounds. Polysaccharides, laetiporic acids, beauvericin, lectins, and triterpenes can be of interest as nutraceuticals and pharmaceuticals (e.g., anti-tumor, anti-HIV, immunomodulation, and hypocholesterolaemic agents (Hwang et al. 2008; Radic et al. 2009; Turkoglu et al. 2007). Moreover, *L. sulphureus* basidiocarps are a rich source of α-(1 → 3)-d-glucan. Their cell wall contains up to 88 % of the dry matter of this glucan, whereas in other fungi it is present in an amount of 9–46 % (Grün et al. 2003).

In the literature, there are no data on the cultivation of *L. sulphureus* fungi on a larger than laboratory scale. Fruiting bodies, in most cases only in the form of primordia, can be obtained in *in vitro* conditions or from long-term cultivation on wood (Ershova et al. 2003; Olennikov et al. 2009).

Our recent studies have shown that *L. sulphureus* fruiting bodies containing large amounts of α-(1 → 3)-glucan can be used as inexpensive and safe raw material to obtain an alternative inducer of α-(1 → 3)-d-glucanases (mutanases, enzymes capable of removing dental and denture plaques) in *Trichoderma harzianum* and *Paenibacillus curdlanolyticus* (Wiater et al. 2008; Pleszczyńska et al. 2010). However, the problem lies in the periodic occurrence the basidiocarps in a natural habitat. This paper is the first report of successful pilot-scale production of mature fruiting bodies of *L. sulphureus* on an artificial sawdust substrate.

## Materials and methods

### Strains

Mycelia of different *L. sulphureus* strains were collected from different species of deciduous trees occurring naturally in some regions of Poland. Pure cultures of 12 strains were obtained by excising pieces of trama from inner parts of carpophores and transferring them onto malt extract agar medium and then onto potato dextrose agar medium. Each incubation was carried out for 14 days at 25 °C. Pure cultures were deposited at the Collection of Edible and Medicinal Mushrooms of the Department of Vegetable Crops of Poznań University of Life Sciences. Mycelium for inoculation of cultivation substrates was prepared on wheat grains using the traditional method (Stamets 2000). Strains were identified by molecular biological analysis of the internal transcribed region (ITS) of the 5.8S rDNA as described below.

### Genomic DNA isolation, amplification of ITS sequences and DNA sequencing

The extraction procedure followed the methods of Borges et al. (1990) with minor modifications. 150-mg portions of lyophilized fruiting body were suspended in a lysis buffer (4 mM spermidine, 10 mM EDTA, 100 mM NaCl, 0.5 % SDS, 10 mM β-mercaptoethanol, 40 mM Tris-HCl, pH 8.0). After incubation at 65 °C for 40 min in an Eppendorf Thermomixer comfort, the samples were sequentially extracted with Tris-buffered phenol containing 0.2 % β-mercaptoethanol and then chloroform, centrifuged for 20 min at 10,000*g*, precipitated with ice-cold ethanol, washed with 70 % ethanol, dried and redissolved in TE buffer (1 mM Tris-HCl, 100 mM EDTA, pH 8.0). The purity and concentration of the DNA samples were evaluated using ND-1000 (Nanodrop, USA). Polymerase chain reaction amplifications (PCR) followed the protocol of White et al. (1990) in a final volume of 50 μL. The primers ITS1, ITS2, ITS3, and ITS4 were used for PCR amplification and sequencing of the internal transcribed spacers from the ribosomal genes. Reactions were performed in a TPersonal thermocycler (Biometra, Germany). Amplified PCR products were quantified by gel electrophoresis on a 1 % agarose gel stained with ethidium bromide and purified by microfiltration using a Clean-up kit (A&A Biotechnology, Poland). Sequencing was performed by fluorescent dye-terminator chemistry with the automated sequencer ABI 3730 (Applied Biosystems Inc., USA) following the manufacturer’s instructions.

### Cultivation experiment

The experimental substrate comprised oak sawdust and a mixture of birch (60 %), alder (20 %), aspen (10 %) and poplar (10 %) sawdust. Oak sawdust and the mixture of sawdust were blended at a ratio of 1:1 by volume. The ratio of small to medium to large (chips) sawdust fraction in the substrate was 3:5:2. In order to optimize the composition of the experimental substrate, it was enriched with an organic and an inorganic additive. The organic additive comprised a mixture of materials of agricultural origin with a composition as shown in Table 1. The inorganic additive was a mixture of mineral salts as collated in Table 2. Each substrate portion was mixed with 7.2 % (in relation to dry weight of substrate) of the mineral additive, supplemented with the organic additives (10, 20, 30, 40 and 45 % dry weight/dry weight of substrate) and the moisture content was adjusted to 40, 50, 55, 60 and 65 %. The prepared substrates were placed in 22 cm × 12 cm × 17 cm polypropylene bags (Mycomed, Poland) with microporous filters. Each bag was shaped into a rectangle block, which contained 1.4 kg dry weight of the substrate. Experimental substrates were sterilized for 8 h at 105–108 °C, then cooled down to 21 °C, and inoculated with 20 g grain spawn per bag. The bags were tightly closed and were subsequently kept in a spawn running room at 23 ± 1 °C and an air humidity of 65–70 %. Incubation was continued until the entire surface of the substrate was colonized by the mycelium.

**Table 1:** Organic supplements mixture

Component	% (w/w)
Wheat bran	35
Rye bran	20
Ground corn	15
Triticale grain	15
Millet grain	10
Buckwheat bran	5

**Table 2:** Mineral salts mixture

Component	g/bag
Gypsum	50
Dolomite	22
Sucrose	15
Chalk	15
Salt solution^a^	120 mL

^a^(NH_4_)_2_SO_4_—5 g, K_2_HPO_4_—5 g, MgSO_4_—0.5 g, and H_2_O to 1,000 g

The following methods were tested for induction of *L. sulphureus* fruiting: (1) injection, through a filter, of 300 mL sterile water at 10 °C into the bag; (2) incubation the bag at 2–4 °C for 24 h; (3) cut-off the top of the bag; (4) incision of the side surfaces of the bag (four cuts, 1 cm long). The bags with the substrate were then kept at 23 ± 1 °C and 65–68 % relative humidity with a 10 h photoperiod. Adequate ventilation was provided to prevent an increase in CO_2_ concentration. Fruiting bodies were harvested from the substrates when mature. Biological efficiency (BE %) was calculated using an equation reported by Stamets (2000) as follows: (fresh weight of harvested fruiting bodies/dry matter content of the substrate) × 100.

In the cultivation experiment, five replications were carried out for each combination of strain, substrate moisture content, level of organic supplementation, and method of fruiting induction. Altogether, a total of 3,000 cultivation units were used.

## Results and discussion

Twelve strains of *Laetiporus* were isolated from a natural habitat. Molecular analysis of the ITS region of the isolated strains clearly identified them as belonging to the genus *L. sulphureus*. The nucleotide sequences have been deposited in the GenBank nucleotide sequence database under Accession No. from HM015201 to HM015212. All the isolated strains (LAE 01–LAE 12) were tested for production of fruiting bodies in a cultivation experiment.

It should be mentioned that, in natural conditions, fruiting bodies of *L. sulphureus* develop on dying trees, mainly during late spring and early summer (Gumińska and Wojewoda 1985) when there is active transfer of water and nutrients between the root zone and tree crowns. Such changes in the wood infested with *L. sulphureus* mycelium can act as factors provoking development of carpophores. Development of carpophores can further be stimulated by climatic conditions, among others, by air humidity as well as temperature differences between day and night. The natural conditions discussed can be difficult to emulate under the conditions of laboratory experiments and commercial production, especially in view of the fact that the stimulation of carpophore formation can be a result of a joint action of these factors.

When designing our research method, attempts were made to take into consideration the above-mentioned factors. A synthetic log production system with sawdust as the main ingredient was chosen for cultivation of the *L. sulphureus* strains. In order to formulate the substrate for the growth of *L. sulphureus* mycelium and fruiting body production, the basal ingredient was enriched with a constant concentration of a mineral additive and an organic supplement used in a wide concentration range from 10 to 45 %. Moreover, on the basis of the preliminary trial, it was concluded that the growth of *L. sulphureus* mycelium significantly depended on the substrate moisture content. The mycelium growth was promoted by a moisture content of 40, 50 and 60 %, lower and higher values resulted in a decrease of growth rate (data not shown). Analogous results have been reported by Habijanič and Berovič (2000) and Wang et al. (2001) who have demonstrated that the substrate moisture content affects growth and yield of different fungi species. Therefore, in the cultivation experiments, it was decided that a substrate moisture content in the range of 40–65 % should be used.

**Fig. 1:**
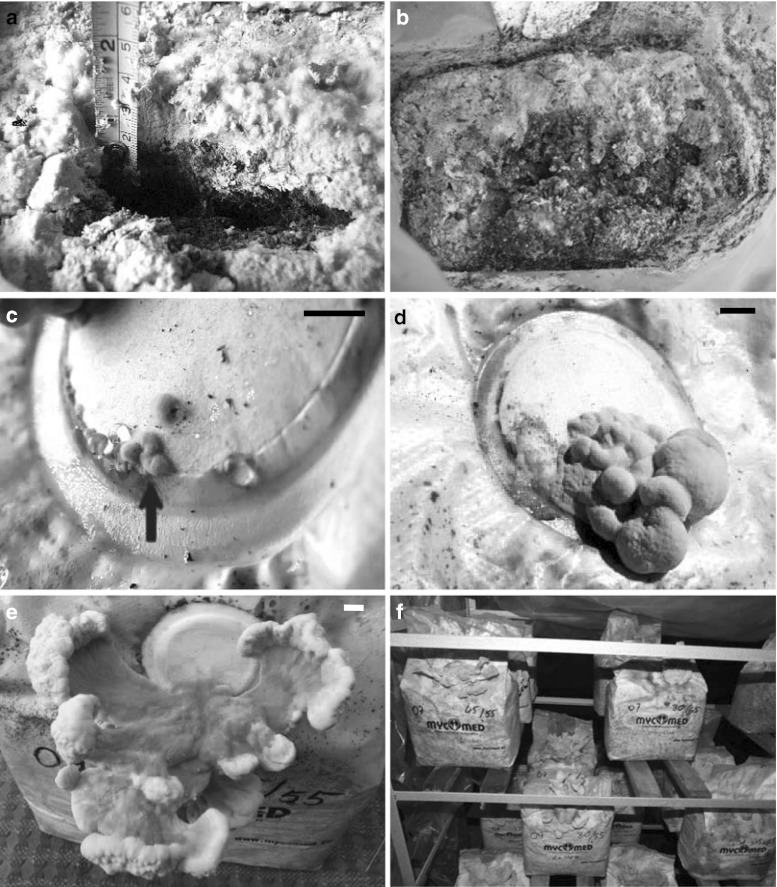
Time course of *L. sulphureus* LAE 01 fruiting body formation under controlled conditions. **a** Mycelial mat produced on the substrate surface (34 days after inoculation); the ruler provides a scale for the size of the mycelial layer. **b** Infection of the substrate surface with *Trichoderma* sp. **c** 5–6-day-old primordia; *red arrow* points to the first primordium. **d** Immature fruiting bodies. **e** Mature fruiting body. **f** Bags with mature fruiting bodies. In each panel, *bars* represent 1 cm. (Color figure online)

It was found that in all the experimental treatments, *L. sulphureus* mycelium colonized the substrate completely after 4 weeks of incubation. The amount of the organic additives applied failed to affect mycelium growth. However, it was observed that the *L. sulphureus* strains examined differed with respect to the speed of substrate colonization by mycelium. The substrate was colonized the fastest by mycelia of the strains LAE 01, LAE 03 and LAE 12. The mycelium of *L. sulphureus* colonized the media in a unique way. As shown by the example of strain LAE 01 (Fig. 1a), it formed a 2-cm thick yellow–orange or yellow–pink dense “skin” of a foamy-gelatinous consistency on the surface of the substrate, while the deeper layers of the substrate were very loosely overgrown by whitish fungal hyphae. Unlike most species of cultivated fungi, which after complete colonization of the substrate constitute a compact structure on the surface and inside the medium of which carpophores develop (Stamets 2000), *L. sulphureus* fails to develop such a thick and tenacious mycelial mat that could be a good base for forming fruiting bodies.

Several methods of fruiting body induction were tested. All the methods that caused the loss of sterility of the substrate in the bag (unsealing the bag through the incision or partial removal of the foil) proved to be useless due to infections of the exposed substrate surfaces with fungi from the genera *Penicillium* and *Trichoderma*. Very soon (within 4–5 days), the mycelia of these fungi overran completely the exposed *L. sulphureus* mycelium (Fig. 1b). The effective methods of induction included injection of a portion of cold water through a microbiological filter or cooling the bag with the substrate at 2–4 °C for 24 h, but better results were achieved using the former method (data not shown). Primordia of *L. sulphureus* could be observed on the surface of filters already within 5–6 days after induction (Fig. 1c), while after another 2 days, the primordia began to develop into fruiting bodies (Fig. 1d–f). Out of the 12 isolates, the formation of fruiting bodies was observed in two strains, i. e. LAE 01 and LAE 12.

**Fig. 2:**
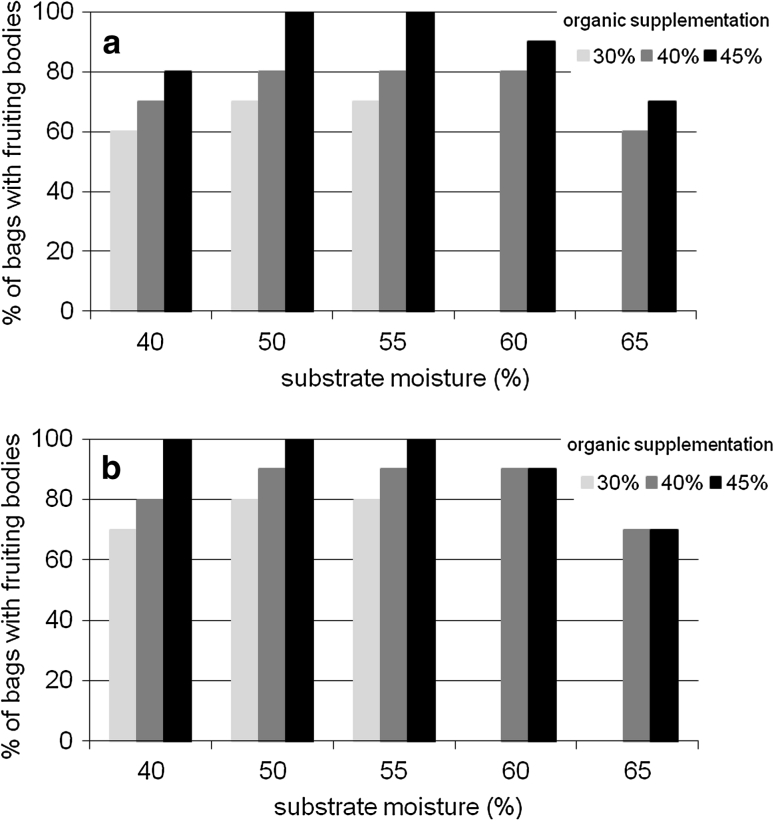
Influence of organic supplementation and substrate moisture content on fruiting body formation by *L. sulphureus*. **a** Strain LAE 01. **b** Strain LAE 12

The levels of organic supplementation turned out to play a crucial role in the process of *L. sulphureus* fructification. Fruiting bodies of the LAE 12 and LAE 01 strains were obtained only on substrates containing at least 30 % of the mixture of organic supplements. However, the number of bags in which carpophores were formed depended on the amount of the applied organic additive. The highest percentage of substrate-filled bags in which there appeared fruiting bodies of both *L. sulphureus* strains was recorded when the supplementation reached 45 %; in the remaining combinations, this proportion was lower. It was further observed that, at this level of organic supplementation, in the case of strain LAE 01, carpophores appeared on all the substrates with a moisture content of 50 and 55 %, while in strain LAE 12—on substrates with a moisture content ranging from 40 to 55 % (Fig. 2a–b).

**Fig. 3:**
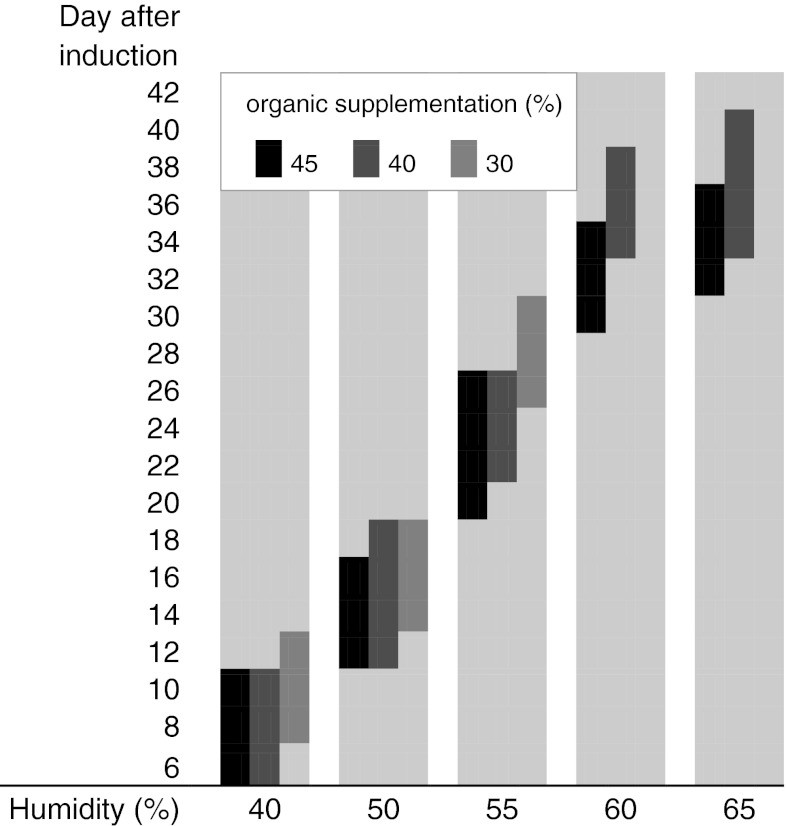
Effect of organic supplementation and water content in the substrate on the fruiting time of *L. sulphureus* LAE 01

As shown by the example of strain LAE 01 (Fig. 3), the time required for the formation of fruiting bodies also depended on the level of organic supplementation and moisture content of the substrate. Fruiting bodies appeared the earliest (after 6 days) on substrates with high organic supplementation (45 and 40 %) and a low water content (40 %). Simultaneously, the higher the moisture of the substrate, the later the fruiting bodies appeared. When the water content reached 60–65 %, fruiting began only after 28–30 days from the date of induction.

In the harvest, fruiting bodies reached a weight of 200–300 g and broke off under their own weight; this prevented statistical analysis of the data and determination of the attainable value of the biological efficiency (BE). For this reason, only one flush was obtained due to the infection at the carpophore fracture site. The BE calculated for the combinations for which fruiting bodies appeared on all the substrates, ranged from 15 to 21 %. It can be assumed that after minor modification of culture technology, larger fruiting bodies could be obtained.

The most important fact arising from the present study was that mature fruiting bodies of *L. sulphureus* could be produced on a large scale on a bed of sawdust under controlled conditions. In the literature, there are only a few studies on procedures for obtaining immature fruiting bodies in *in vitro* conditions. Ershova et al. (2003) obtained *L. sulphureus* fruiting bodies of a spherical shape and a diameter of 5–8 cm in Erlenmeyer flasks. Fruiting bodies grew on the surface of agar medium supplemented with rye flour for more than 3 months of incubation. In another experiment, fruiting bodies of *L. sulphureus* were chamber-grown on a sterile sunflower shell substrate at 21 °C, without low-temperature stimulation. Primordia appeared about 14 days after the substrate was fully colonized (Olennikov et al. 2008).

The natural plantation method of growing *L. sulphureus* has also been reported (Olennikov et al. 2009). Larch (*Latrix sibirica* Ledeb.) wood was used as a substrate. After inoculation with the agar mycelium, the substrate was incubated in a constant-temperature cabinet at 21 °C. The stumps overgrown with mycelium were then transferred to experimental plantations in Pribaikal’e (Irkutsk region). However, also in this case there is no direct evidence that the method allows to obtain fully mature fruiting bodies.

In conclusion, we provide the first report of successful large-scale production of mature fruiting bodies of *L. sulphureus* on an artificial substrate. The strains of *L. sulphureus* used in this study were isolated from a natural habitat and grew on enriched sawdust. Two of the strains fruited successfully. High levels of organic supplementation of the substrate promoted fruiting body formation and simultaneously reduced the time required for fruiting to 6 days after induction. The optimum moisture content of the substrate was 40 %. An increase in the water content resulted in prolonged fruiting run time. Our future studies will focus on further optimization of cultivation conditions of *L. sulphureus* and improving the technology of fruiting body production to increase the yield and biological efficiency of the fungi.
